# MiR-192-5p inhibits proliferation, migration, and invasion in papillary thyroid carcinoma cells by regulation of SH3RF3

**DOI:** 10.1042/BSR20210342

**Published:** 2021-09-24

**Authors:** Songbo Fu, Chengxu Ma, Xulei Tang, Xiaoni Ma, Gaojing Jing, Nan Zhao, Juntao Ran

**Affiliations:** 1 The First Clinical Medical School, Lanzhou University, Lanzhou, Gansu 730000, China; 2 Department of Endocrinology, The First Hospital of Lanzhou University, Lanzhou, Gansu 730000, China; 3 Department of Radiation Oncology, The First Hospital of Lanzhou University, Lanzhou, Gansu 730000, China

**Keywords:** microRNA-192-5p, papillary thyroid cancer, SH3RF3

## Abstract

**Background:** The decreased level of miR-192-5p has been reported in several kinds of cancers, including bladder, colon, ovarian, and non-small cell lung cancer. However, the expression and function of miR-192-5p in papillary thyroid carcinoma/cancer (PTC) remains unknown.

**Objective:** The present study aimed to explore the function and underlying mechanism of miR-192-5p in PTC development.

**Methods:** PTC tissues and relative normal controls from PTC patients were collected. qRT-PCR analysis was performed to measure miR-192-5p and SH3RF3 mRNA level in PTC tissues and cell lines. CCK-8 method and FCM assay were used to test cell proliferation and apoptosis in TPC-1 cells, respectively. The abilities of cell migration and invasion were detected by wound healing and transwell assays, respectively. The protein expression was evaluated by Western blot. The interaction between miR-192-5p and Src homology 3 (SH3) domain containing ring finger 3 (SH3RF3) were confirmed by dual-luciferase reporter assay.

**Results:** MiR-192-5p level was obviously decreased in PTC tissues and cell lines. Overexpression of miR-192-5p suppressed proliferation, migration, invasion, and EMT process, while induced apoptosis in TPC-1 cells. In addition, miR-192-5p negatively modulated SH3RF3 expression by binding to its 3′-untranslated region (3′UTR). Silencing SH3RF3 inhibited the migration, invasion, and EMT of TPC-1 cells. In the meantime, matrine, an alkaloid extracted from herb, exerted its anti-cancer effects in PTC cells dependent on increase in miR-192-5p expression and decrease in SH3RF3 expression.

**Conclusion:** We firstly declared that miR-192-5p played a tumor suppressive role in PTC via targeting SH3RF3. Moreover, matrine exerted its anti-cancer effects in PTC via regulating miR-192-5p/SH3RF3 pathway.

## Introduction

Papillary thyroid carcinoma/cancer (PTC) is the most prevalent subtype of thyroid cancer, occupying more than 75% of all thyroid cancer cases. Most PTC patients are curable and display excellent prognosis after treating with surgery and radioactive iodine. However, a certain proportion of patients with PTC were unresponsive to the current therapies and exhibited relapses, or even developed to advanced or distantly metastatic PTC [1]. Hence, it is urgent to further investigate the molecular mechanism of PTC to develop new alternative therapeutic agents.

Since the first microRNA (miRNA) identified in 1993, research on miRNA biology has grown rapidly and accumulating miRNAs were discovered to play crucial roles in various biological processes, such as cell differentiation, proliferation, and survival [2,3]. In addition, aberrant miRNAs expression was found in numerous diseases, especially in cancer. MiRNAs could act as either oncogenes or tumor suppressors by binding to 3′-untranslated region (3′UTR) of their targeted genes, leading to mRNA degradation or transcriptional suppression, thus regulating tumor development [4–7]. For example, miRNA-21, a tumor suppressor, promoted breast cancer proliferation and metastasis by down-regulating leucine zipper transcription factor-like 1 [8]. Due to the modulation of tumor initiation and progression, miRNAs have become novel markers for diagnosis and attractive therapeutic targets for some tumors [9–11].

Several studies have demonstrated that miR-192-5p was remarkably decreased in a spectrum of human tumors, such as bladder cancer [12], colon cancer [13], ovarian cancer [14], and non-small cell lung cancer [15]. Moreover, miR-192-5p expression was abundant in normal liver tissues but significantly down-regulated in cancer stem cell (CSC)-positive HCC tissues. Reduced miR-192-5p led to hepatic carcinogenesis by enhancing CSC populations and CSC-associated properties [16]. Nevertheless, the expression of the functions of miR-192-5p in PTC is still unknown.

SH3RF3 (SH3 domain containing ring finger 3, also known as plenty of SH3 domains protein 2) is a newly discovered protein with four Src homology 3 (SH3) domains and a Ring finger domain [17]. SH3RF3 can function as a scaffold interacting with PAK2 and RAC1 via its SH3 domain [18,19]. Moreover, its ring finger domain also confers a self-ubiquitination enzymatic activity to the protein [20]. Analysis of clinical samples indicated that SH3RF3 level was correlated with several kinds of disease including breast cancer [21]. Moreover, the high expression of SH3RF3 in tumor tissues was correlated with the increases CSC number and weak prognosis of breast cancer. However, the role of SH3RF3 in PTC needs to be further clarified.

In the present study, we found miR-192-5p expression level was significantly decreased in PTC tissues and cell lines. MiR-192-5p overexpression suppressed proliferation, migration, and invasion, as well as EMT process in TPC-1 cells. Mechanistically, miR-192-5p suppressed the malignant activities of PTC cells by negatively regulating SH3RF3. Meantime, matrine, a potential agent for treating PTC, exerted its anti-cancer effects dependent on up-regulating of miR-192-5p expression.

## Materials and methods

### PTC samples collection

A total of 20 pairs of matched PTC species and relative non-tumorous tissues were obtained from patients after removal of primary PTC at our hospital from 2016 to 2018. All patients had written the informed consents and the present study was approved by the Ethics Committee of our hospital.

### Cell culture and transfection

Human thyroid follicular epithelial cell line Nthy-ori 3-1 and human PTC cell line TPC-1 were reserved in our laboratory. Cryopreserved cells were recovered from liquid nitrogen using an AccuVital cell recovery kit (AccuRef Scientific, Xi’an, China) and cultured in RPMI-1640 complete culture medium. The other two human PTC cell lines BHT101 and B-CPAP were acquired from Cell Bank of Type Culture Collection (Guangzhou, China) and cultured in Dulbecco’s modified Eagle’s complete medium and grown at 37°C with 5% CO_2_.

MiR-192-5p inhibitors, mimics, siRNA specific for SH3RF3, pcDNA3.1-SH3RF3, and relative negative controls (NCs) were synthesized by Sangon (Shanghai, China). Cell transfection in the present study was performed by utilizing Lipofectamine 2000 (Invitrogen, U.S.A.) according to standard procedures.

### *qRT*-*PCR*

Total RNA was isolated from PTC tissues and cell lines using an RNA isolation kit (AccuRef Scientific) and cDNA was synthesized by a FastQuan RT Kit (TIANGEN, China) with random primers. qRT-PCR was performed on a Bio-Rad Real-Time PCR System, using SYBR Green MasterMix (Thermo Scientific). Primer sequences are listed in Table 1.

**Table 1:** The sequences of PCR primers

Gene	Sequences
miR-192-5p	F: 5′-GGACTTTCTTCATTC ACACCG-3′
	R: 5′-GACCACTGAGGTTAGAGCCA-3′
SH3RF3	F: 5′-CGGAATTCATGCTGCTCGGAGCGTCCTGGCTG-3′
	R: 5′-CGGGATCCTCTCAGAAGCTCTCGACGAAG-3′
E-cadherin	F: 5′-GGCACAGATGGTGTGATTAC-3′
	R: 5′-GAGCACC TTCCATGACAGA-3′
Vimentin	F: 5′-ATGACCGCTTCGCCAACTAC-3′
	R: 5′-CGGGCTTTGTCGTTGGTTAG-3′
Fibronectin	F: 5′-TGCTGGGACTTCCTACGT- CG-3′
	R: 5′-CGTTTGAGTTGCCACCGTAAG-3′
β-actin	F: 5′-CTTCCTGGGCATGGAGTC-3′
	R: 5′-GCCGATCCACACGGAGTA-3′

### Cell proliferation assay

TPC-1 cells were planted in a 96-well plate (2 × 10^3^ per well) and each group included six replicates. After maintaining for indicated time in cell incubator, CCK-8 (AccuRef Scientific) was supplemented and the absorbance at 450 nm of the culture was tested.

### Cell apoptosis assay

Forty-eight hours after transfection, TPC-1 cells were harvested, washed with pre-cooled PBS, and then incubated with propidium iodide (PI) and fluorescein isothiocyanate (FITC)-labeled Annexin V on ice for 15 min. Then, flow cytometry (BD Biosciences, U.S.A.) was utilized to detect the cell apoptosis rate.

### Wound healing assay

The ability of cell migration was evaluated by the wound healing assay. TPC-1 cells were planted into 24-well plates at a density of 2 × 10^5^/well. When cell adherence reached to 90%, a 200-μl tip was utilized to scratch the wound. At indicated time, the healing of scratches in each well was photographed and the relative migration distance was calculated as compared with control group.

### Cell invasion assay

Boyden chamber (BD, U.S.A.) was used to test the invasion ability of TPC-1 cells. The chambers with 8.0-µm membrane were placed into 24-well plates. Cells in serum-free RPMI-1640 medium were adjusted to a concentration of 2 × 10^5^/ml. Then 500 µl of the cell suspension and 600 µl complete medium supplemented with 10% FBS were added to the upper chambers pre-coated with Matrigel and lower chambers, respectively. Twenty-four hours later, the cells under the transwell membrane were stained with Crystal Violet and counted.

### Prediction of miR-192-5p potential downstream targets

Downstream targets of miR-192-5p were predicted by the online databases of PicTar (https://pictar.mdc-berlin.de/), microT (http://diana.imis.athena-innovation.gr/DianaTools/index.php?r=mrmicrot/index), TargetScan (http://www.targetscan.org/vert_72/), and miRDB (http://mirdb.org/index.html) with their online default parameters. Then, the overlapped targets were screened using Venn analysis (version 2.1, https://bioinfogp.cnb.csic.es/tools/venny/).

### Dual luciferase reporter assay

The wildtype (WT) or mutant (MUT) fragment of SH3RF3 3′UTR was obtained and inserted into pmirGLO vectors (Promega, U.S.A.). Then, TPC-1 cells were co-transfected with miR-192–5p mimics or inhibitor and the reporter vector SH3RF3-WT or SH3RF3-MUT. Forty-eight hours post-transfection, dual luciferase reporter assay system (Promega, U.S.A.) was performed to determine the luciferase activity and collection.

### Western blot analysis

Cells were lysed by RIPA buffer (Exprecision, Xi’an, China) containing with proteinase inhibitors (1 mM PMSF) on ice. Quantification of protein was performed by using a BCA protein quantification kit (AccuRef Scientific). Equal amounts of proteins were subjected to SDS/PAGE and transferred to PVDF membrane (Millipore, U.S.A.). The membrane was incubated with primary antibodies and secondary antibody (1:2000, Abcam) sequentially, and then detected by ECL detection reagents (Exprecision). Primary antibodies against E-cadherin (1:1000, Abcam), vimentin (1:1000, Abcam), fibronectin (1:3000, Proteintech™), and SH3RF3 (Thermo Fisher, 1:200) were used in the present study. β-actin (1:1000, Abcam) was used as an internal control.

### Statistical analysis

GraphPad Prism version 7.0 was employed for statistical analysis and data were presented with mean ± standard deviation. The differences between groups were compared by analysis of variance tests or Student’s *t* tests. Each experiment was repeated independently at least three times. *P*<0.05 was regarded as the level of significant difference.

## Results

### The level of miR-192-5p was decreased in PTC tissues and cell lines

**Figure 1:**
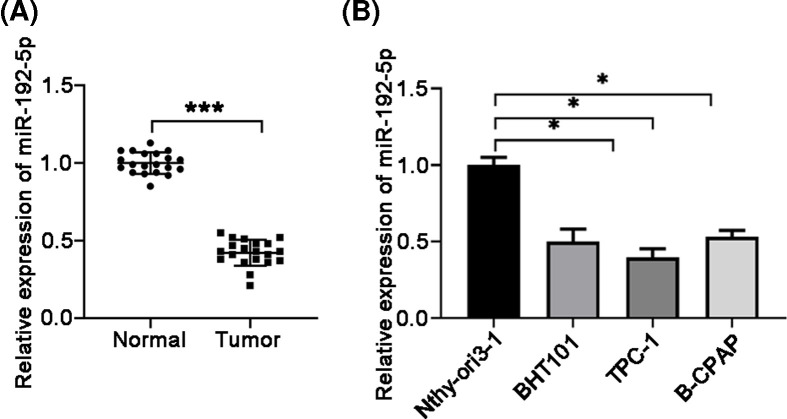
Relative expression levels of miR-192-5p in PTC tissues and cell lines (**A**) miR-192-5p expression in 20 pairs of human PTC tissues and adjacent normal tissues detected by qRT-PCR. Compared with the Normal group, ***P<0.001. (**B**) Detection of miR-192-5p expression levels in PTC cell lines (BHT101, B-CPAP, and TPC-1) and in the normal thyroid follicular epithelial cell line Nthy-ori3-1 by qRT-PCR. Compared with the Nthy-orl3-1 cells, *P<0.05.

To explore the role of miR-192-5p in PTC, 20 pairs of human PTC tissues and corresponding normal tissues were collected and qRT-PCR analysis was used to determine the expression level of miR-192-5p. As shown in Figure 1A, expression of miR-192-5p was significantly decreased in PTC tissues compared with adjacent normal tissues. In addition, miR-192-5p expression was also measured in human thyroid follicular epithelial cell line Nthy-ori 3-1 and PTC cell lines BHT101, B-CPAP, and TPC-1. The results showed that miR-192-5p level was decreased dramatically in three PTC cell lines compared with the normal control cell line (Figure 1B). Taken together, these data suggested that miR-192-5p was probably involved in PTC development.

### MiR-192-5p overexpression inhibited PTC cell proliferation and enhanced apoptosis

**Figure 2:**
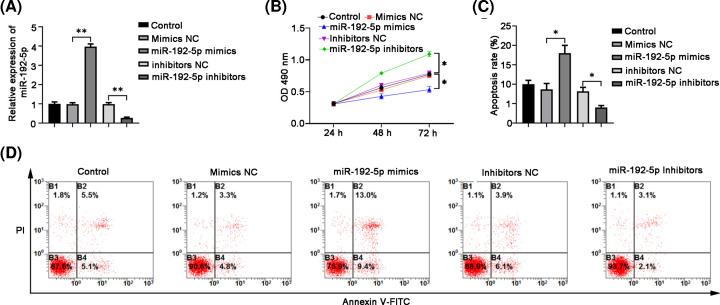
MiR-192-5p overexpression significantly suppresses the proliferation and induces cell apoptosis in TPC-1 cells (**A**) qRT-PCR analysis validated the effects of miR-192-5p mimics and inhibitors on its expression in TPC-1 cells. (**B**) CCK-8 assay was performed to evaluate cell proliferation. (**C**) Quantification of apoptosis determined by flow cytometry. (**D**) Flow cytometry with Annexin V-FITC/PI double staining to detect TPC-1 apoptosis. Compared with the NC group, *P<0.05 and **P<0.01.

To evaluate the functions of miR-192-5p in cell proliferation and apoptosis of PTC cells, we modulated miR-192-5p expression by transfecting TPC-1 cells with miR-192-5p mimics, inhibitor, or corresponding NCs. As shown in Figure 2A, qRT-PCR results showed that miR-192-5p expression displayed an increase by over 3.5-fold in the miR-192-5p mimics group, while a decrease by 5-fold in the miR-192-5p inhibitors group, compared with their corresponding NC groups. Then CCK-8 and FCM assays were performed to test the effects of the abnormal miR-192-5p expression on cell proliferation and apoptosis, respectively. As shown in Figure 2B, transfection of miR-192-5p mimics significantly suppressed TPC-1 cell proliferation compared with mimic NC. Instead, transfection of miR-192-5p inhibitors enhanced TPC-1 cell proliferation compared with inhibitor NC. FCM data revealed that the apoptosis rate in TPC-1 cells exhibited an increase in miR-192-5p mimics group, while a decrease in miR-192-5p inhibitors group (Figure 2C).

### MiR-192-5p overexpression suppressed cell migration, invasion, and EMT

**Figure 3:**
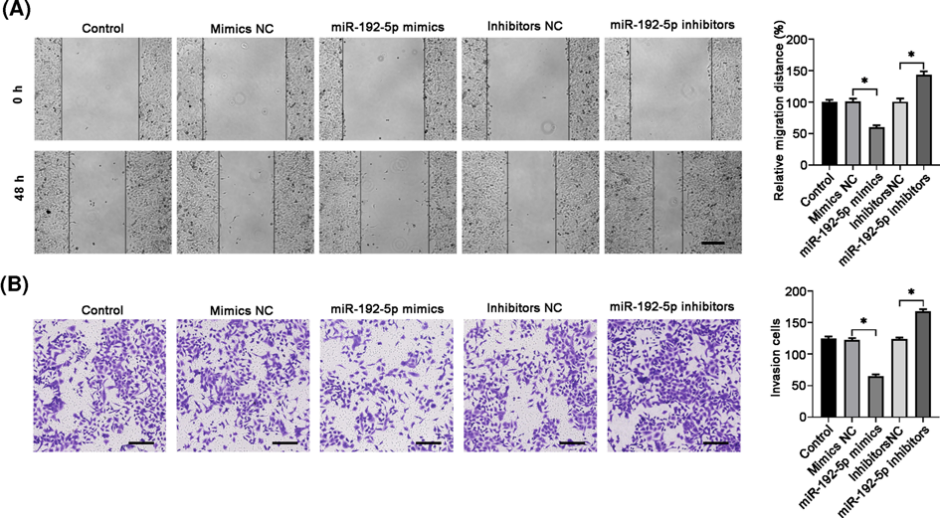
Overexpression of miR-192-5p suppresses TPC-1 cell migration and invasion (**A**) Wound-healing assay was performed to detect TPC-1 cell migration. (**B**) Transwell invasion assay was performed to evaluate cell invasion. Compared with the NC group, *P<0.05.

**Figure 4:**
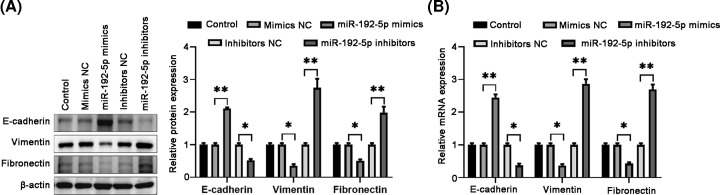
Overexpression of miR-192-5p suppresses EMT in TPC-1 cells (**A**) EMT-related proteins E-cadherin, vimentin, and Fibronectin were evaluated by Western blotting. (**B**) The mRNA expressions of the EMT-associated factors E-cadherin, vimentin, and Fibronectin were measured by using qRT-PCR. Compared with the NC group, *P<0.05 and **P<0.01.

Then, we tested the effects of miR-192-5p on TPC-1 cell migration and invasion. Wound-healing analysis showed that miR-192-5p overexpression significantly reduced cell migration, while miR-192-5p knockdown led to an increase in cell migration, compared with the corresponding NC control (Figure 3A). In addition, cell invasion ability displayed a decline in TPC-1 cells transfection with miR-192-5p mimics, while an augment in miR-192-5p inhibitors group (Figure 3B). The above data indicated that miR-192-5p might influence the EMT process. Therefore, we further measured the EMT-associated markers expression in TPC-1 cells. Western blotting results showed that miR-192-5p mimics enhanced the epithelial marker E-cadherin level and reduced the mesenchymal marker vimentin and fibronectin expression. On the contrary, miR-192-5p inhibitors exhibited the opposite effects (Figure 4A). The mRNA level of E-cadherin, vimentin and fibronectin showed similar trends in TPC-1 cells after knocking down or overexpressing miR-192-5p (Figure 4B). These data suggested that miR-192-5p inhibited EMT process in PTC cells.

### MiR-192-5p directly targeted SH3RF3

**Figure 5:**
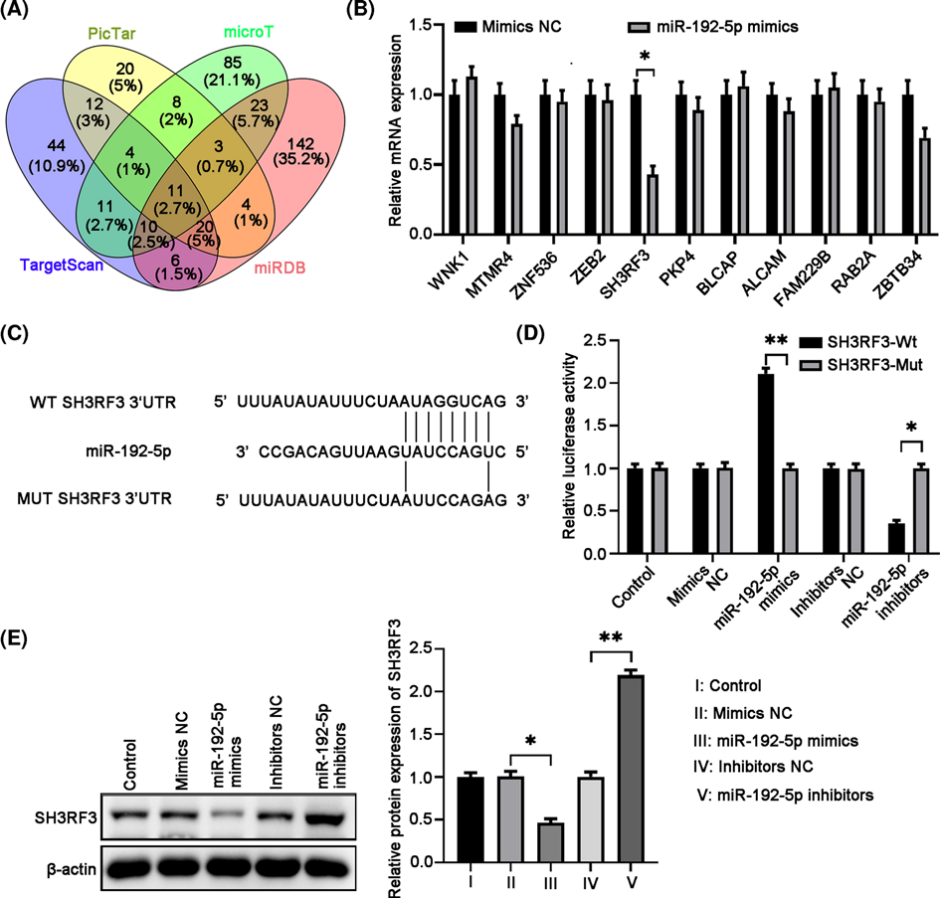
*SH3RF3* gene is a direct target of miR-192-5p (**A**) MiR-192-5p interacted with SH3RF3 was predicted by using bioinformatics online tools. (**B**) TPC-1 cells were transfected with miR-192-5p mimics or relative control, the expression of predicted target genes was detected by qRT-PCR. (**C**) The binding sites for miR-192-5p and SH3RF3 are indicated. (**D**) Luciferase reporter assay was used to confirm the direct binding between miR-192-5p and SH3RF3. (**E**) The SH3RF3 protein expression in TPC-1 cells transfected with miRNA mimics or inhibitors by Western blot was detected. Compared with the indicated groups, *P<0.05 and **P<0.01.

To explain the regulatory effects of miR-192-5p on PTC cell malignant activities, we intend to search for the downstream targets for miR-192-5p. Bioinformatics analysis was performed to predict the target genes and 11 genes were returned (Figure 5A). TPC-1 cells were transfected with miR-192-5p mimics and all the predicted target mRNAs expression were detected by qRT-PCR. Among the 11 mRNAs, the level of SH3RF3 was the lowest one (Figure 5B). The 3′UTR of SH3RF3 containing the sequence complementary to the miR-192-5p was shown in Figure 5C. Then dual luciferase reporter assays were conducted to testify whether miR-192-5p targeted SH3RF3 by binging to its 3′UTR. Our results showed that miR-192-5p remarkably decreased the luciferase activity of a SH3RF3-3′UTR WT reporter plasmid, whereas miR-192-5p inhibitors increased it. Interestingly, both miR-192-5p mimics and inhibitors did not affect the luciferase activity of SH3RF3-3′UTR mutant reporter (Figure 5D). Western blot analysis results showed that miR-192-5p mimics reduced while miR-192-5p inhibitors increased SH3RF3 protein expression in TPC-1 cells (Figure 5E). These results suggested that SH3RF3 was a direct target of miR-192-5p.

### Silencing SH3RF3 inhibited TPC-1 cell migration, invasion, and EMT

**Figure 6:**
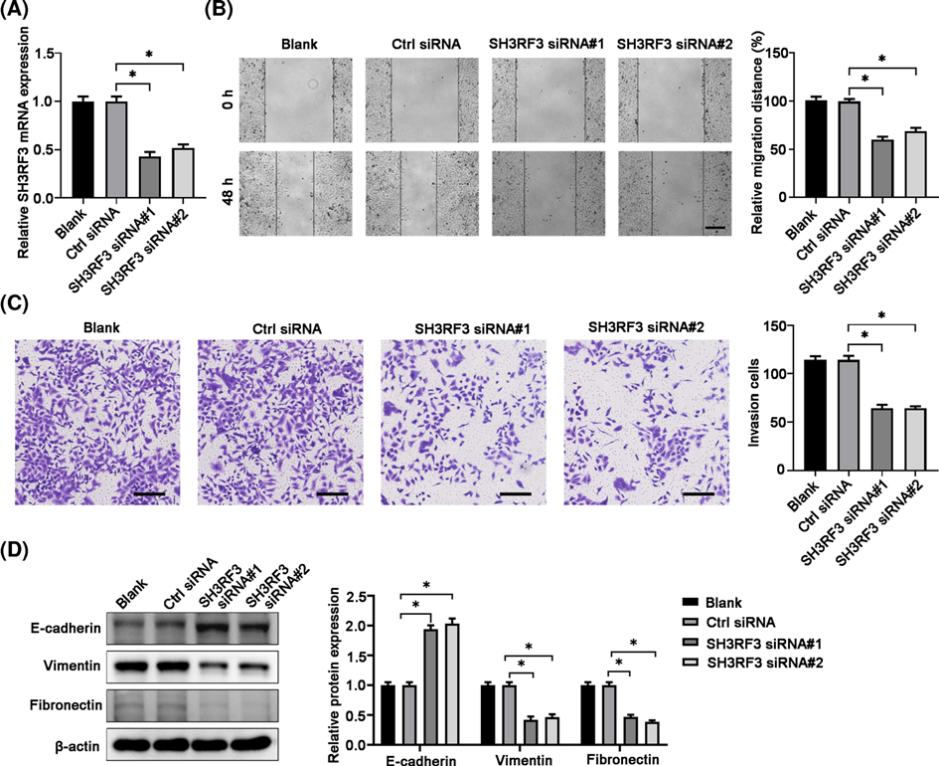
SH3RF3 knockdown inhibits TPC-1 cell migration, invasion, and EMT (**A**) Transfection efficiency of the SH3RF3 siRNAs was evaluated by qRT-PCR. (**B**) Wound healing assay revealed that SH3RF3 siRNA decreased TPC-1 cell migration. (**C**) Transwell assay revealed that SH3RF3 siRNA decreased cell invasion. (**D**) Protein expression levels of EMT-related proteins E-cadherin, vimentin, and Fibronectin were analyzed by using Western blot in various experimental conditions. Compared with the indicated groups, *P<0.05.

Since SH3RF3 was a direct target gene of miR-192-5p, we further evaluated its function in TPC-1 cells via transfection with siRNAs specifically target to SH3RF3 to down-regulate its expression. Two siRNAs were designed and synthesized and the SH3RF3 knockdown effect was shown in Figure 6A. Wound-healing assay results displayed that SH3RF3 knockdown suppressed cell migration in TPC-1 cells compared with blank and control siRNA groups (Figure 6B). Cell invasions were also significantly reduced by silencing SH3RF3 (Figure 6C). Moreover, SH3RF3 knockdown led to an increased expression of E-cadherin, while a decreased expression of vimentin and fibronectin (Figure 6D).

### Overexpression of miR-192-5p reverses the effect of SH3RF3 in promoting migration and invasion of TPC-1 cells

**Figure 7:**
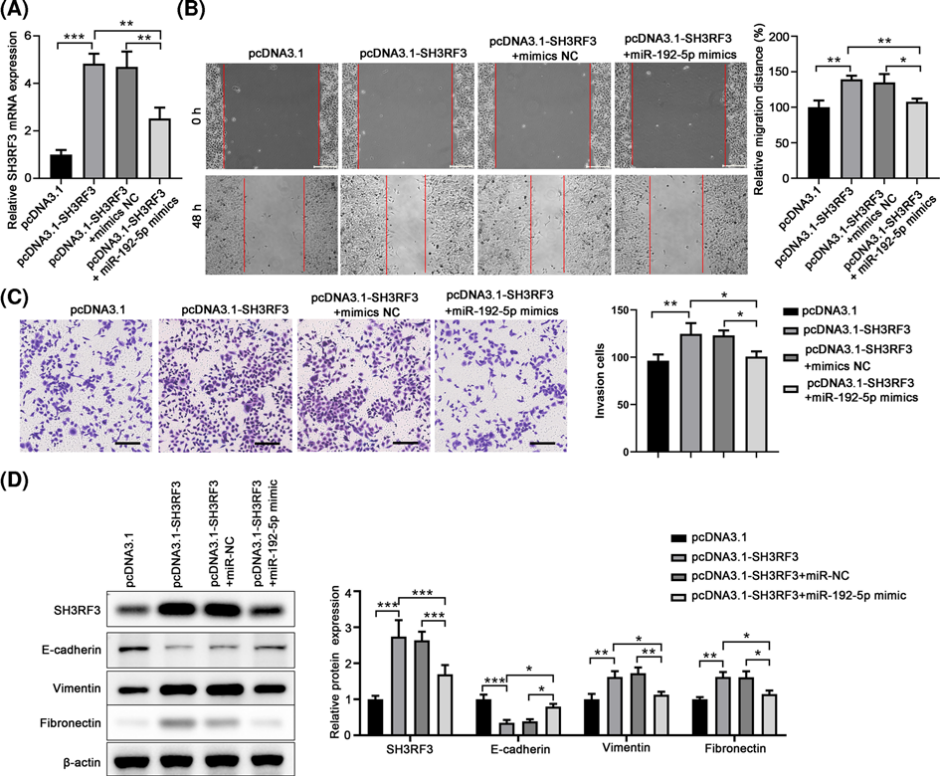
miR-192-5p reverses the effect of SH3RF3 in promoting the migration, invasion, and EMT of TPC-1 cells (**A**) Transfection efficiency of the pcDNA3.1-SH3RF3 and/or miR-192-5p was evaluated by qRT-PCR. (**B**) Wound healing assay revealed that the effect of SH3RF3 and/or miR-192-5p on the migration of TPC-1 cells. (**C**) Transwell assay revealed that the effect of SH3RF3 and/or miR-192-5p on the invasion of TPC-1 cells. (**D**) Protein expression levels of EMT-related proteins E-cadherin, vimentin, and Fibronectin were analyzed by using Western blot in various experimental conditions. Compared with the indicated group, *P<0.05, **P<0.01, and ***P<0.001.

To further confirm the regulatory relationship between miR-192-5p and SH3RF3, SH3RF3 and miR-192-5p were co-transfected into TPC-1 cells followed by migration and invasion analyses. qRT-PCR analysis showed that overexpression of SH3RF3 significantly increase SH3RF3 expression, while overexpression of miR-192-5p mimics markedly reversed this up-regulation (Figure 7A). Wound healing and transwell assays presented that overexpression of SH3RF3 obvious promoted the migration and invasion of TPC-1 cells, while overexpression of miR-192-5p mimics remarkably aborted these enhancement effects of SH3RF3 (Figure 7B,C). In addition, Western blot assay showed that overexpression of SH3RF3 obviously decreased E-cadherin expression but increased vimentin and fibronectin expression, while overexpression of miR-192-5p mimics significantly reversed these alterations induced by SH3RF3 (Figure 7D). These findings suggested that miR-192-5p might inhibit the migration and invasion of TPC-1 cells via targeting SH3RF3.

### Matrine suppressed TPC-1 cell migration and invasion by regulating the miR-192-5p/SH3RF3 pathway

**Figure 8:**
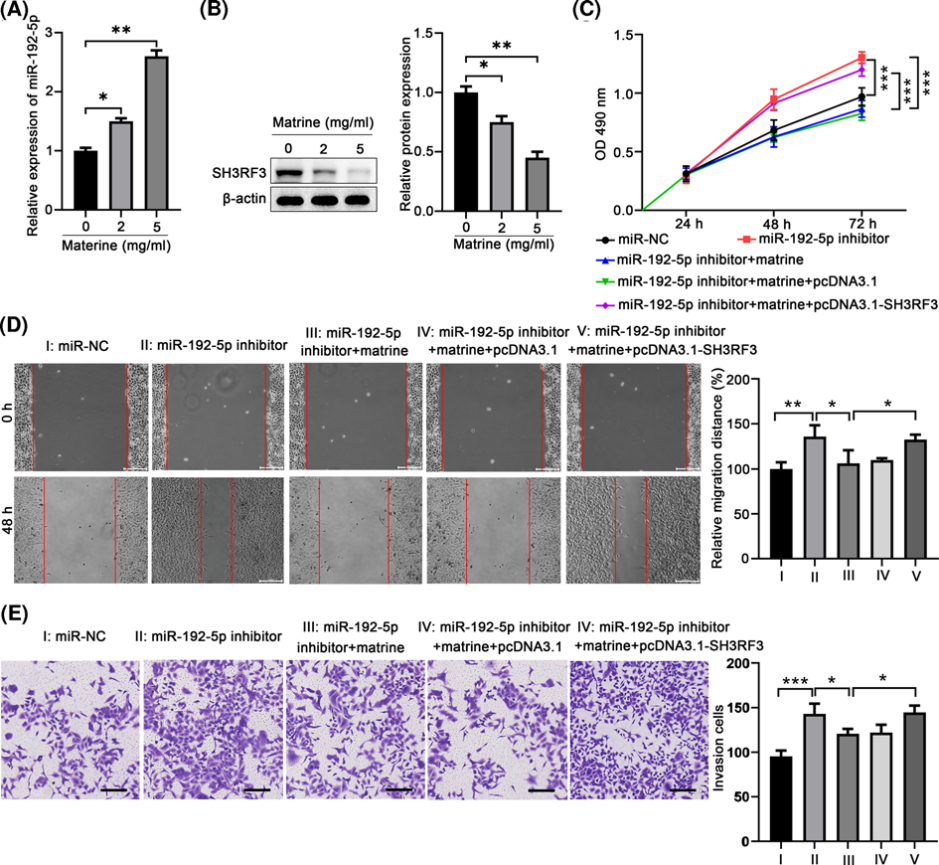
Matrine treatment suppresses human thyroid cancer cell migration and invasion by regulating the miR-192-5p/SH3RF3 pathway (**A**) Analysis of miR-192-5p expression levels in PTC cell lines following treatment with 0, 2, 5 mg/ml matrine by qRT-PCR. (**B**) Analysis of SH3RF3 protein expression level in PTC cell lines followed 0, 2, 5 mg/ml matrine treatment by Western blot. (**C**) CCK-8 assay evaluated the proliferation of PTC cells after matrine treatment (5 mg/ml) in the presence or absence of miR-192-5p inhibitors or and pcDNA3.1-SH3RF3. (**D**) The influence of matrine treatment (5 mg/ml) on cell migration ability was analyzed by wound healing assay in the presence or absence of miR-192-5p inhibitors or and pcDNA3.1-SH3RF3. (**E**) Transwell invasion assay was used to evaluate cell invasion ability after matrine treatment (5 mg/ml) in the presence or absence of miR-192-5p inhibitors or and pcDNA3.1-SH3RF3. Compared with the indicated group, *P<0.05, **P<0.01, and ***P<0.001.

Matrine has been reported to be a potential agent for treating human PTC. Therefore, we further investigated whether the anti-cancer effects of matrine was correlated with the miR-192-5p/SH3RF3 pathway. Firstly, we found that matrine significantly up-regulated miR-192-5p expression (Figure 8A) and down-regulated SH3RF3 protein expression (Figure 8B) in TPC-1 cells in a dose-dependent manner. Further analyses revealed that matrine could markedly inhibit the proliferation, migration, and invasion of miR-195 inhibitors overexpressed TPC-1 cells, but overexpression of SH3RF3 markedly reverses the effect of matrine to promote the proliferation, migration, and invasion of TPC-1 cells (Figure 8C–E). These findings indicated that the matrine could inhibit the proliferation, migration, and invasion of PTC via modulating miR-192-5p/SH3RF3 pathway.

## Discussion

Although most PTC patients showed favorable prognosis after comprehensive treatment, some of the patients suffer from the recurrence and metastases of PTC [22]. Therefore, further exploring the deep mechanism of PTC progression and searching for novel targets for PTC therapy are strongly needed. A number of miRNAs were found to be abnormally expressed in a broad spectrum of cancers, acting as an oncogene or tumor suppressor, and modulated cancer cell proliferation, apoptosis, migration, and invasion by negatively controlling the expression of their targets [2,7]. Some miRNAs even could be biomarkers for cancer diagnosis and prognostic, as well as therapeutic targets. As far as PTC is concerned, some kinds of miRNAs are also reported. For example, serum levels of miR-222, miR-221, and miR-146b functioned as good biomarker for PTC diagnosis and predicted its progression and recurrence [23]. Therefore, looking for deregulated miRNA and understanding the molecular mechanisms regulating the development of PTC will be helpful in identifying novel diagnostic, prognostic, and therapeutic targets.

Evidence has demonstrated the role and function of miR-192-5p in several types of cancer, including lung, colon, bladder, breast and liver cancer. For example, miR-192-5p inhibited lung cancer bone metastasis by negatively modulating TRIM44 expression [24]. Li et al. demonstrated that miR-192-5p restrained bladder cancer growth via targeting Yin Yang 1 [12]. In addition, the sensitivity of breast cancer cells to doxorubicin could be enhanced by miR-192-5p [25]. However, a recent research demonstrated that miR-192-5p promoted the proliferation and metastasis in hepatocellular carcinoma cell [26]. Therefore, whether miR-192-5p served as an oncogene or a tumor suppressor in PTC, it remains largely unknown. In the present study, we found that the level of miR-192-5p was significantly declined in PTC tissues and three PTC cell lines including BHT101, B-CPAP, and TPC-1. Moreover, miR-192-5p overexpression remarkably suppressed cell proliferation, migration, invasion and EMT process, while induced cell apoptosis in TPC-1 cells. These findings indicated that miR-192-5p played a tumor suppressive role in PTC, which consisted with previous studies.

Seeking out the direct target bound to miRNAs could be helpful in clarifying the deep molecular mechanisms of the regulatory role of miRNAs. Several mRNA targets of miR-192-5p have been reported, such as peptidylprolyl isomerase A [25], ERCC3/ERCC4 [27], and TRIM44 [24]. By bioinformatics analysis, 3′UTR of SH3RF3 was predicted to have complementary sequence to miR-192-5p and dual luciferase reporter assays confirmed the direct binding between miR-192-5p and SH3RF3. Moreover, miR-192-5p mimic decreased, while miR-192-5p inhibitor increased the expression of SH3RF3 in TPC-1 cells. Then we further evaluated the functions of SH3RF3 in PTC. Our data showed that SH3RF3 silencing significantly suppressed the migration, invasion and EMT process, indicating that SH3RF3 served as on oncogene in PTC. Our results were in line with a recent report which demonstrated that SH3RF promoted breast cancer progression by enhancing JNK phosphorylation and the activation of the JNK-JUN pathway. Therefore, our findings suggested that miR-192-5p regulated PTC in an SH3RF-dependent manner.

Matrine, an alkaloid extracted from the herb root, has been reported to display anti-tumor functions against a variety of cancers including PTC [28]. However, the deep mechanisms of its anti-tumor effects are not clear. Hence, we hypothesized that matrine exerted its tumor suppressive role by regulating miR-192-5p/SH3RF axis in PTC. Our results showed that matrine could enhanced miR-192-5p expression, while decreased SH3RF3 level in a dose-dependent manner. Moreover, miR-192-5p inhibitor could partially reverse the suppressive effects of matrine on cell proliferation, migration, and invasion in TPC-1 cells, suggesting the anti-tumor effects of matrine is fulfilled partially through modulating miR-192-5p/SH3RF pathway.

In summary, it is the first study to declare that miR-192-5p played a tumor suppressive role in PTC cells via modulating SH3RF3 expression. In addition, matrine exerted anti-tumor effects in PTC via influencing miR-192-5p/SH3RF pathway. Therefore, our findings indicate that targeting miR-192-5p/SH3RF axis may shed light on PTC treatment.

## Data Availability

The datasets generated and/or analyzed during the present study are available from the corresponding author on reasonable request.

## Abbreviations

CCK-8: cell counting kit-8
CSC: cancer stem cell
EMT: epithelual-mesenchymal transition
FCM: Flow cytometry
FITC: fluorescein isothiocyanate
miRNA: microRNA
NC: negative control
PI: propidium iodide
PTC: papillary thyroid carcinoma/cancer
qRT-PCR: quantitative real time polymerase chain reaction
SH3: Src homology 3
SH3RF3: SH3 domain containing Ring finger protein 3
WT: wildtype
3′UTR: 3′-untranslated region
